# Angiogenesis is associated with the onset of hyperplasia in human ductal breast disease

**DOI:** 10.1038/sj.bjc.6605196

**Published:** 2009-07-21

**Authors:** J E Bluff, S R Menakuru, S S Cross, S E Higham, S P Balasubramanian, N J Brown, M W Reed, C A Staton

**Affiliations:** 1 Microcirculation Research Group, Academic Unit of Surgical Oncology, University of Sheffield Medical School, Sheffield, South Yorkshire, UK; 2 Academic Unit of Pathology, University of Sheffield Medical School, Sheffield, South Yorkshire, UK

**Keywords:** angiogenesis, VEGF, tissue factor, HIF-1*α*, breast cancer, hyperplasia

## Abstract

**Background:**

The precise timing of the angiogenic switch and the role of angiogenesis in the development of breast malignancy is currently unknown.

**Methods:**

Therefore, the expression of CD31 (pan endothelial cells (ECs)), endoglin (actively proliferating ECs), hypoxia-inducible factor-1 (HIF-1*α*), vascular endothelial growth factor-A (VEGF) and tissue factor (TF) were quantified in 140 surgical specimens comprising normal human breast, benign and pre-malignant hyperplastic tissue, *in situ* and invasive breast cancer specimens.

**Results:**

Significant increases in angiogenesis (microvessel density) were observed between normal and benign hyperplastic breast tissue (*P*<0.005), and between *in situ* and invasive carcinomas (*P*<0.0005). In addition, significant increases in proliferating ECs were observed in benign hyperplastic breast compared with normal breast (*P*<0.05) cancers and in invasive compared with *in situ* cancers (*P*<0.005). Hypoxia-inducible factor-1*α*, VEGF and TF expression were significantly associated with increases in both angiogenesis and proliferating ECs (*P*<0.05). Moreover, HIF-1*α* was expressed by 60–75% of the hyperplastic lesions, and a significant association was observed between VEGF and TF in ECs (*P*<0.005) and invasive tumour cells (*P*<0.01).

**Conclusions:**

These findings are the first to suggest that the angiogenic switch, associated with increases in HIF-1*α*, VEGF and TF expression, occurs at the onset of hyperplasia in the mammary duct, although the greatest increase in angiogenesis occurs with the development of invasion.

Breast cancer is the most frequently diagnosed cancer in women in both Europe and the United States, with an estimated 608 380 new cases of invasive disease in 2007 (Ferlay *et al*, 2007). Breast cancer is a genetically heterogeneous disease, and in contrast to the clearly defined pathway from normal through adenomatous to invasive malignancy associated with increasing dysplasia in colorectal cancer, there is considerable uncertainty about the pathogenesis of different forms of breast cancer and the relationship between hyperplasia and carcinoma of the breast (Wiechmann & Kuerer, 2008). However, there is good evidence of an increased risk of breast cancer associated with some hyperplastic lesions (for example, atypical ductal hyperplasia; ADH) and clear evidence of progression from *in situ* to invasive disease, particularly in high-nuclear-grade lesions, although at a relatively low rate, for example, 5–10% over 10 years (Jones, 2006).

Many cancer patients display a hypercoagulable state leading to complications, such as deep vein thrombosis and pulmonary embolism (reviewed by Bluff *et al*, 2008). Upregulation of tissue factor (TF), the primary initiator of the coagulation cascade, has a key role in this process (Molnar *et al*, 2007). In normal physiological conditions, TF, a 47 kDa transmembrane protein, is constitutively expressed by subendothelial cells (such as pericytes, smooth muscle cells and fibroblasts), whereas vascular endothelial cells (ECs) and intravascular cells do not express TF (Abe *et al*, 1999). Initiation of the extrinsic coagulation pathway occurs when TF is exposed to the bloodstream, either after damage to the normal integrity of the vascular EC lining or on activation of monocytes or ECs. Factor VIIa (FVIIa) then binds to TF on the cell surface. Sequential downstream activation of haemostatic protease complexes leads to the generation of thrombin, with subsequent platelet activation and the formation of a fibrin clot, which restores vessel integrity (reviewed by Rak *et al*, 2006).

During tumourigenesis, the strict regulation of TF expression is lost. Upregulation of TF protein by tumour cells and associated stromal cells has been well documented in breast cancer and other malignant tumours, and may contribute to tumour angiogenesis, metastasis, hypercoagulability and tumour cell survival (reviewed by Bluff *et al*, 2008). Furthermore, correlations of elevated TF expression with advanced stages of malignancy have been reported in different cancers, including colon (Nakasaki *et al*, 2002) and breast (Ueno *et al*, 2000), suggesting that TF may have a role in tumour progression. In addition, TF is known to contribute to tumour progression indirectly, through the role in haemostasis, and, in part, by upregulating the pro-angiogenic vascular endothelial growth factor-A (VEGF) (Zhang *et al*, 1994; Abe *et al*, 1999). VEGF, a 34–45 kDa protein, is the most potent known stimulator of angiogenesis (the outgrowth of new capillaries from an existing vascular bed), an essential process in tumour growth, progression and metastasis (Folkman, 1995). Although TF has the potential to induce VEGF expression, the primary driving force for angiogenesis is oxygen concentration, and VEGF is known to be upregulated by hypoxia-inducible factor-1 (HIF-1*α*), in regions of hypoxia within solid tumours (Mizukami *et al*, 2007).

The stage at which angiogenesis occurs in tumour progression is known as the ‘angiogenic switch’ (Folkman *et al*, 1989). Angiogenesis in invasive breast cancer is well documented (Bos *et al*, 2005), but relatively few studies have addressed the role of angiogenesis in pre-malignant ductal disease or where the angiogenic switch occurs during the development of breast malignancy. This study therefore quantifies angiogenesis and the number of proliferating ECs in addition to the expression of HIF-1*α*, VEGF and TF in hyperplastic lesions, *in situ* and invasive breast carcinomas, to determine whether angiogenesis may have a role in dysplastic transformation.

## Materials and methods

### Patients

Archival histological specimens were obtained from 140 patients at the Royal Hallamshire Hospital between 1995 and 2003 with approval from the Local Research Ethics Committee (Ethics number SSREC 98/137). Haematoxylin and eosin sections of all specimens were reviewed by SSC (a consultant histopathologist) and these consisted of (a) normal breast tissue from patients undergoing breast reduction surgery (*n*=8); (b) benign hyperplastic breast tissue (usual ductal hyperplasia; UDH (*n*=30)); (c) ADH (*n*=27); (d) *in situ* cancer (ductal carcinoma *in situ* (DCIS); low/intermediate nuclear grade (*n*=19)); (e) high-nuclear-grade DCIS (*n*=25); and (f) invasive breast cancer specimens (invasive ductal carcinoma; IDC; (*n*=31)). Breast cancer patients were excluded from the study if they received neo-adjuvant treatment.

### Immunohistochemistry

Formalin-fixed (10% formaldehyde), paraffin-embedded breast specimens were sectioned (5*μ*m) and mounted on 3-aminopropyltriethoxysilane (Sigma Aldrich, Dorset, UK)-coated slides. Immunohistochemistry was performed, at working concentrations of 10–25*μ*g ml^−1^, using a panel of antibodies to human platelet EC adhesion molecule-1 (PECAM-1/CD31; 20*μ*g ml^−1^) (M0823; Dakocytomation Ltd, Cambridgeshire, UK), endoglin (CD105; 25*μ*g ml^−1^) (M3527; Dakocytomation Ltd), HIF-1*α* (610959 BD Pharmingen, Oxford, UK; 20*μ*g ml^−1^), VEGF (A-20; Santa Cruz Biotechnology, Wiltshire, UK; 10*μ*g ml^−1^) and TF (4509; America Diagnostica Inc, CT, USA; 20*μ*g ml^−1^). A standard horseradish peroxidase staining procedure was followed using an appropriate biotinylated secondary antibody (Vector laboratories, Peterborough, UK) at 5–10*μ*g ml^−1^, followed by the elite ABC kit (Avidin : Biotinylated enzyme Complex; Vector laboratories) and DAB (3, 3′diaminobenzidine) as the chromogen substrate (Vector laboratories). All sections were counterstained with Gill's haematoxylin (Sigma Aldrich) to visualise cell nuclei. For HIF-1*α* staining, antigen retrieval was performed by heating sections at 95°C for 45 min in target retrieval solution (Dakocytomation Ltd). For all other antibodies, the same antigen retrieval was performed: sections were heated in a microwave in TRIS/EDTA for 3 min on high power, followed by 7 min on low power. Appropriate normal sera and casein, both diluted 1 : 10 in phosphate-buffered saline, were used for blocking all sections and dilution of antibodies. Samples known to be positive for each factor (wounds and invasive breast cancer tissue) were included in each staining run. Negative controls were achieved by replacement of the primary antibody with mouse IgG_1_-irrelevant antibodies. Furthermore, specificity of the VEGF antibody was confirmed by pre-absorption with a blocking peptide to VEGF (sc-152P; Santa Cruz Biotechnology). A recombinant TF protein (2339-PA; R & D Systems, Oxfordshire, UK) was used as a pre-absorption control for TF immunostaining because no blocking peptides were available for this factor.

### Microvascular density and proliferating EC assessment

Microvascular density (MVD), a surrogate marker of angiogenesis, was assessed using the Chalkley grid method, as described previously (Vermeulen *et al*, 2002). Briefly, the tissue was screened at low magnification ( × 40) for the five most microvascular dense areas within each stained specimen (‘vascular hotspots’) that were <150 *μ*m from any adjacent lesion. Subsequently, each vascular hotspot was viewed at higher magnification ( × 400) using a Chalkley grid graticule, which has 25 randomly placed dots. Under high power, the dots were aligned to touch the maximum number of vessels and these were counted. This resulted in a Chalkley grid score for each hotspot; the sum of the Chalkey scores for each of the five hotspots was termed the cumulative Chalkley score (CCS). CD31 staining was used to assess MVD; endoglin staining (CD105) was used to assess the number of proliferating ECs in each breast specimen.

### Quantification of HIF-1*α*, VEGF and TF staining

All analyses were carried out by an assessor fully trained by a consultant histopathologist (SSC). HIF-1*α* staining was considered to be positive when localised to the nuclei of >10% of ductal epithelial cells (Chen *et al*, 2005). Pure cytoplasmic staining was considered to be negative. Vascular endothelial growth factor-A staining, if present in a lesion, was observed in >90% of cells; this was assessed semi-quantitatively using a grading system, which reflected the intensity of staining present within the specimen (0=no staining; 1=weak staining; 2=moderate staining; and 3=strong staining). In contrast, HIF-1*α* and TF staining were graded as present or absent, because no obvious differences in the staining intensity of these factors were observed in the different specimens examined. An invasive breast cancer specimen was regarded as positive for TF expression when staining was identified in >10% of the tumour cells in the section.

### Intra- and interobserver error

The reproducibility of the Chalkley grids method for quantifying MVD/proliferating ECs, HIF-1*α*, VEGF and TF staining was established by at least 10% of the slides being counted by a second experienced observer in a blinded manner. A selection of the slides was also counted for a second time by the first observer. Intra- and interobserver error was assessed 4 weeks apart, on 20 slides from each immunohistochemically stained group and analysed statistically.

### Follow-up

Follow-up information, including the date and cause of death, were gathered by written enquires to the Trent Cancer Registry, according to the ethics committee approval. Patients who had died during the follow-up period from cancer-unrelated causes were treated as censored by statistical survival analysis.

### Statistical analysis

Statistical analysis was conducted using SPSS v15 software (SPSS Inc. Chicago, IL, USA). Appropriate non-parametric tests were used to investigate protein expression (Mann–Whitney U-test for two independent groupings, Kruskal–Wallis test for more than two independent groupings and Jonckheere–Terpstra test for ordinal categorical groupings). If continuous data were not normally distributed, correlations were analysed with Spearman's correlation statistics. Survival curves were plotted using the Cox regression model and analysis was carried out using ‘death due to breast cancer’ as the end point for overall survival. The influence of MVD and number of proliferating ECs was assessed using the Cox proportional hazards regression model. Data were considered statistically significant at the level of *P*<0.05.

## Results

### Assessment of angiogenesis

**Figure 1:**
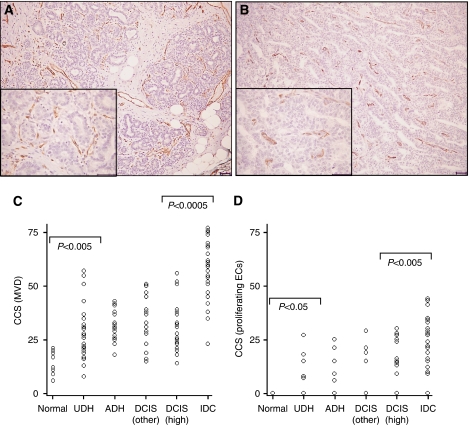
Endothelial staining in breast lesions. Immunohistochemical staining of ECs with (**A**) CD31 and (**B**) endoglin in high-grade DCIS. Note the increased frequency and intensity of stained ECs for CD31 compared with endoglin (scale=50 *μ*m). Scatter plots representing cumulative Chalkley scores in different breast tissue samples immunostained with (**C**) CD31 (MVD) and (**D**) endoglin (proliferating ECs). (**C**) There was a significant increase in MVD between normal breast tissue and UDH (*P*<0.005), and a further significant increase in MVD between *in situ* (DCIS) and invasive breast cancers (*P*<0.0005). (**D**) There was a significant increase in proliferating ECs in UDH cases compared with normal breast (*P*<0.05) and in IDC compared with high-grade DCIS specimens (*P*<0.005). *P*<0.05 was considered significant (Kruskal–Wallis followed by Mann–Whitney U-test).

Strong CD31 staining was observed along the cell membrane of ECs in all the breast specimens observed (Figure 1A). The median CD31 MVD count was 14.5 in normal breast (range, 6–21; Figure 1C), which increased significantly to 28 in benign hyperplastic tissue (UDH) (range, 13–57; *P*<0.005). Microvascular density scores were constant in UDH/ADH breast samples and in *in situ* cancers of all grades, but there was a further significant increase in MVD between *in situ* and invasive carcinomas, with a median MVD count of 31 (range, 14–56) in high-grade DCIS and 59 (range, 23–74) in IDC (*P*<0.0005).

The immunostaining of vessels with CD31 (pan ECs) exhibited an increased frequency and intensity, when compared with endoglin, in all types of breast tissues (Figure 1A and B). In contrast to CD31 staining, endoglin was not expressed in normal breast ECs, but was expressed by ECs in 17% of UDH cases, 19% of ADH cases, 21% and 44% of DCIS cases (low/intermediate grade and high grade, respectively) and in 84% of invasive carcinomas (Figure 1D). Moreover, there was a significant correlation between vessels staining for both endoglin and CD31 (Spearman's rho correlation coefficient=0.516; *P*<0.005). The correlation coefficients for inter- and intraobserver error scores for MVD and proliferating ECs were 0.91 and 0.86, and 0.92 and 0.89, respectively, showing a high level of agreement.

### Assessment of HIF-1*α*

**Figure 2:**
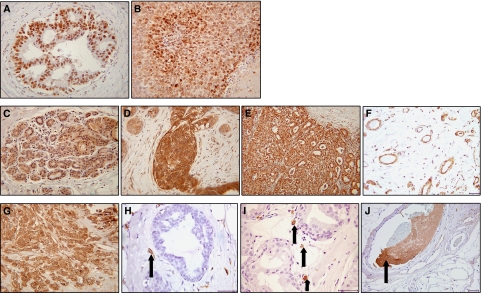
Immunohistochemical staining for HIF-1*α*, VEGF and TF. (**A**) Nuclear HIF-1*α* staining in the ductal epithelial cells of an ADH case and (**B**) tumour cells of an invasive cancer. (**C**) Weak expression of VEGF in normal breast epithelium (score=1), (**D**) strong expression in florid usual ductal hyperplasia (score=2/3) and (**E**) strong staining localised to tumour cells within invasive breast carcinomas (score=3). (**F**) VEGF expression in ECs in normal breast tissue. (**G**) Tumour cells expressed TF in approximately 55% of invasive breast cancer specimens. (**H**) TF was expressed in ECs associated with benign hyperplastic tissue (arrow). (**I**) Putative macrophages expressing TF associated with areas of DCIS (arrows). (**J**) TF expressed in vessel containing thrombosis (arrow). Photographs **A**–**E** and **G** were taken at × 20 magnification and all others at × 40 magnification.

**Figure 3:**
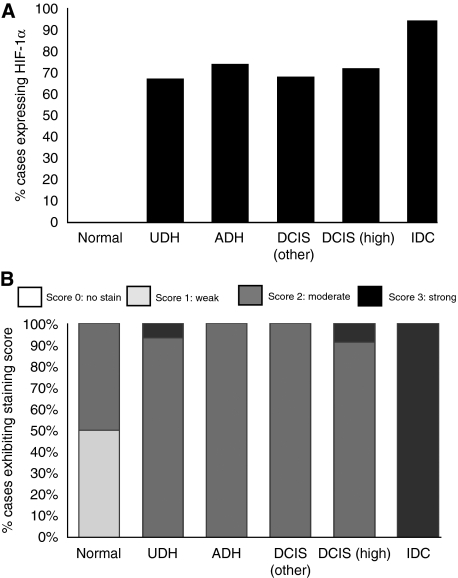
Expression of HIF-1*α* and VEGF in the epithelial/tumour cells of breast lesions. (**A**) Percentage of cases expressing HIF-1*α*. There is a significant increase in HIF-1*α* expression seen with increasing severity of lesion (*P*<0.01) (**B**) Percentage of cases with varying VEGF expression. There is a significant increase in VEGF expression seen with increasing severity of lesion (*P*<0.001).

Hypoxia-inducible factor-1*α* protein was not expressed in normal breast tissue, but was expressed in the nuclei of ductal epithelial cells in 65–75% of hyperplastic breast/*in situ* cancers (Figures 2A and 3A) and in over 90% of invasive cancers (Figures 2B and 3A). There was a moderate correlation between HIF-1*α* expression and MVD (Spearman's rho correlation coefficient=0.502, *P*=0.001) and proliferating ECs (Spearman's rho correlation coefficient=0.301, *P*=0.001). The correlation coefficients for inter- and intraobserver error scores for HIF-1*α* assessment were 0.88 and 0.91, respectively, showing a high level of agreement.

### Assessment of VEGF

Vascular endothelial growth factor-A was expressed in the cytoplasm of ductal epithelial cells in 100% of breast specimens, with weak and/or moderate expression in normal breast tissues (Figures 2C and 3B), with moderate to strong expression in hyperplastic tissue/*in situ* cancers (Figures 2D and 3B) and with strong expression localised to invasive tumour cells (Figures 2E and 3B). Vascular endothelial growth factor-A was also constitutively expressed in breast ECs of normal, hyperplastic and DCIS specimens (Figure 2F), but in contrast to VEGF staining in epithelial cells, there was no change in the EC VEGF intensity in different breast tissue types. Interestingly, most ECs within invasive carcinomas did not express VEGF, whereas stromal cells, such as macrophages and fibroblasts, (based on nuclear and cellular morphology analysis) expressed VEGF in all the different classes of breast tissue examined.

There was a strong correlation between VEGF expression and MVD (Spearman's rho correlation coefficient=0.731, *P*=0.001) and proliferating ECs (Spearman's rho correlation coefficient=0.576, *P*=0.001), and a moderate correlation between VEGF expression and HIF-1*α* (Spearman's rho correlation coefficient=0.317, *P*=0.001) in epithelial/tumour cells. The correlation coefficients for inter- and intraobserver error scores for VEGF assessment were 0.85 and 0.90, respectively, showing a high level of agreement.

### Assessment of TF

In contrast to the constitutive expression of VEGF in ductal epithelial cells, TF was not expressed in normal/hyperplastic breast epithelial cells or in *in situ* cancer cells, but interestingly, TF was expressed by tumour cells in approximately 55% of invasive carcinomas (Figure 2G). Tissue factor was not expressed by ECs in normal breast tissue, but was expressed by some ECs in hyperplastic tissue and in ECs associated with *in situ* cancer (Figure 2H). Similar to the pattern of VEGF staining within the intratumoural vasculature, ECs within invasive cancers rarely expressed TF. Putative macrophages (based on nuclear morphology analysis) expressed TF in all breast tissue types, except normal breast (Figure 2I), and TF expression was also observed in thrombosed vessels within IDC specimens (Figure 2J). The latter were used as an internal positive control for TF immunostaining.

Moderate correlation was seen between TF expression and MVD (Spearman's rho correlation coefficient=0.431, *P*=0.001) and proliferating ECs (Spearman's rho correlation coefficient=0.403, *P*=0.001), and a strong correlation between TF and VEGF expression (Spearman's rho correlation coefficient=0.593, *P*=0.001) in epithelial/tumour cells. In contrast, there was no correlation between TF and HIF-1*α* expression in epithelial/tumour cells. The correlation coefficients for inter- and intraobserver error scores for TF assessment were 0.89 and 0.93, respectively, showing a high level of agreement.

### Survival analysis

**Figure 4:**
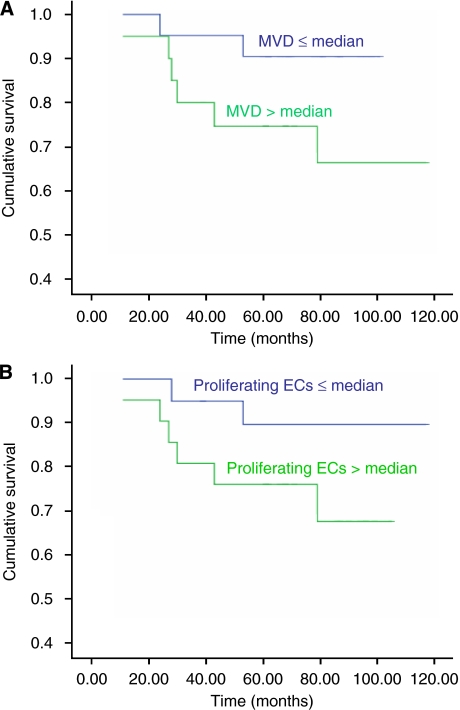
Cox regression survival graphs. Comparison of overall survival between groups of patients with ⩽median and >median cumulative Chalkley scores (CCS) for (**A**) MVD (CD31; *P*=0.09) and (**B**) proliferating ECs (endoglin; *P*=0.12).

All patients with invasive breast cancer at the time of diagnosis were followed up for a median of 71 months (range, 24–117 months). The Cox regression curve for MVD and proliferating ECs (Figure 4) showed a decreased survival in invasive breast cancer patients exhibiting a high CCS (greater than the median (59 for MVD and 22 for proliferating ECs)) when compared with patients with a low CCS (equal to or less than median for both parameters), which did not achieve significance (*P*=0.09 and *P*=0.12, respectively), probably because of the relatively small numbers of patients in the study.

## Discussion

The process of developing a high-density vascular network that connects the tumour and host circulation, termed the ‘angiogenic switch,’ is a crucial step for the progression of a tumour from a benign to malignant state (Folkman *et al*, 1989). Although it has been suggested that the initiation of angiogenesis occurs simultaneous to invasion (reviewed by Bergers & Benjamin, 2003), only one previous study has investigated angiogenesis at the individual stages of the hyperplasia, *in situ* and invasive breast carcinoma spectrum (Pavlakis *et al*, 2008). We show that angiogenesis is significantly increased early in this sequence, confirming this recent data, although the greatest increase occurs with tumour invasion, with associated increases in HIF-1*α*, VEGF and TF expression. Our data therefore suggest that angiogenesis is initiated at the start of hyperplasia, with further increases between *in situ* and invasive carcinomas. Angiogenesis thus starts with hyperplasia before there is any morphological evidence of atypia.

Endoglin has been reported to be highly expressed in the vasculature of various tumour types, including invasive breast carcinomas (Wang *et al*, 1994; Beresford *et al*, 2006), and predicts poor survival and poor clinical response to chemotherapy (Charpin-Taranger *et al*, 2003; Dales *et al*, 2004; Beresford *et al*, 2006). To our knowledge, endoglin has not previously been assessed in each stage of the hyperplasia, *in situ* and invasive breast carcinoma sequence. The significant increase in the percentage of specimens expressing endoglin in both high-grade *in situ* and invasive carcinomas indicates increases in actively proliferating vessels participating in angiogenesis. In agreement with other studies (El-Gohary *et al*, 2007), we identified a significant correlation between vessels stained with endoglin and CD31. However, in this study, between one and two-thirds of the vessels within pre-malignant, preinvasive and invasive breast cancers exhibited proliferating ECs (based on CCS calculated with CD31 and endoglin staining) in contrast to a report by Wang *et al* (1994), which showed that endoglin stained only 20% of the vessels highlighted by CD31 in invasive breast carcinomas.

Previous studies have showed that a high MVD significantly predicts poor survival of breast cancer patients (both relapse-free survival and overall survival) (Uzzan *et al*, 2004) as does a high expression of endoglin (Charpin-Taranger *et al*, 2003; Dales *et al*, 2004). However, in this study, although patients with a high MVD (>59) and high number of proliferating ECs (>22) tended to have a poorer prognosis than patients with a low MVD (⩽59) and low number of proliferating ECs (⩽22) to the same order of magnitude as reported by the previous studies, these did not achieve significance. This may be because of the relatively small number of invasive cancer patients in this study, which was not primarily designed to address this issue.

In addition, a positive correlation was observed between HIF-1*α* and MVD, and between HIF-1*α* and proliferating ECs (based on endoglin expression). Endoglin expression associated with hypoxia within solid tumours has been reported to be directly due to HIF-1*α* binding to the hypoxia response element in the endoglin promoter (Bos *et al*, 2005). As expected, we showed a significant association between HIF-1*α* and VEGF in both ECs and epithelial/tumour cells, as it is known that the HIF-1 complex recognises a consensus hypoxia response element in the promoter of VEGF that mediates hypoxic signals including angiogenesis. Much less expected was the high percentage of hyperplastic breast lesions that expressed HIF-1*α*, as a recent investigation reported that HIF-1*α* was not expressed in hyperplastic lesions (Hao *et al*, 2007). Although hypoxia is one of the most potent stimulators of VEGF, interestingly, not all of the *in situ* and invasive cancers expressed HIF-1*α*. Accumulating evidence indicates that HIF-1-independent pathways can also control angiogenesis (Mizukami *et al*, 2004). Knockdown of HIF-1*α* through small interfering RNA in a colon cancer cell xenograft reduced tumour growth, but surprisingly did not inhibit tumour angiogenesis (Mizukami *et al*, 2005). The specific molecular mechanisms that underlie HIF-1-independent regulation of VEGF are only now being elucidated, but the RAS oncogene seems to have a pivotal role (Ryan *et al*, 2000; Mizukami *et al*, 2004).

Although VEGF is a key mediator of angiogenesis in breast cancer and it is well recognised that VEGF is upregulated in invasive breast carcinomas (Borgstrom *et al*, 1999), much less is known about VEGF expression in hyperplastic and preinvasive lesions. Indeed, although VEGF expression has recently been reported in hyperplastic epithelium (Pavlakis *et al*, 2008), this study is the first to show that this is an upregulation compared with normal breast tissue. The constitutive expression of VEGF in normal breast endothelium may be related to the role of VEGF in preventing apoptosis, which promotes EC survival (Gerber *et al*, 1998), although it is not yet clear how the breast vasculature remains quiescent in the presence of VEGF. Vascular endothelial growth factor-A expression has previously been shown to be significantly associated with MVD in breast cancer (Bolat *et al*, 2006; Pavlakis *et al*, 2008). Moreover, the positive correlations seen in this study between VEGF expression and both MVD and proliferating ECs in hyperplasia, *in situ* and invasive breast carcinoma spectrum indicates that VEGF has a key role in breast cancer angiogenesis, most likely by binding to VEGF receptors on ECs and inducing EC proliferation (Ferrara *et al*, 2003).

The upregulation of TF in invasive breast cancers may be related to the role of TF in metastasis (Bromberg *et al*, 2001; Amarzguioui *et al*, 2006; Ngo *et al*, 2007). Tissue factor is highly expressed in metastatic, but not in non-metastatic breast carcinoma cells (Bluff *et al*, 2006), and may contribute to the metastatic process directly through the TF-FVIIa complex and/or through downstream generation of active coagulation factors (Booden *et al*, 2004; Palumbo *et al*, 2007). Furthermore, TF-FVIIa signalling via PAR-2 has been shown to stimulate breast cancer cell migration (Jiang *et al*, 2004; Morris *et al*, 2006), and a humanised anti-TF monoclonal antibody (CNTO 859) inhibited experimental *in vivo* lung metastasis from invasive breast cancer cells by more than 99%, indicating a role for TF in breast cancer cell metastasis (Ngo *et al*, 2007).

The cytoplasmic domain of TF seems to be essential for the production of VEGF in human gastric cancer (Zhang *et al*, 2005) and melanoma (Abe *et al*, 1999) cells. A positive feedback mechanism induces upregulation of VEGF on tumour cells, further increasing TF expression (Zhang *et al*, 1994). In addition, VEGF has also been reported to induce TF expression in ECs, possibly through early growth response gene-1 and sp1 transcription factors (Armesilla *et al*, 1999; Mechtcheriakova *et al*, 1999). In agreement with these studies, both TF and VEGF were expressed by vessels at the earliest stages of dysplasia and by ECs associated with *in situ* cancers in this study. However, interestingly, both VEGF and TF were rarely expressed by vessels within invasive carcinomas. This may be because of the fact that the plethora of angiogenic molecules increases with malignant progression (reviewed by Folkman, 2007), rendering the tumour less dependent on EC-derived VEGF or TF. Our data suggest that, although VEGF and TF are significant factors in promoting angiogenesis, other molecules, for example, fibroblast growth factor-2 and platelet-derived growth factor, may also have a significant role after downregulation of VEGF and TF within intratumoural vessels (upon tumour invasion).

This is the first report, to our knowledge, that significantly correlates TF expression with MVD and proliferating ECs in the hyperplasia, *in situ* and invasive breast carcinoma spectrum. The association between VEGF and TF in ECs suggests a role for both factors in tumour angiogenesis. Furthermore, the association between VEGF and TF in invasive tumour cells, potentially indicates an intimate relationship in breast cancer disease progression, as has been previously shown (Ueno *et al*, 2000). In addition, macrophages are a major population of infiltrating cells in the tumour stroma; it is possible that VEGF and TF-expressing macrophages, observed in this study, may have a role in regulating the angiogenic switch and tumour progression in human breast cancer, as previously described in an autochthonous mouse model of breast cancer (Lin *et al*, 2006).

In conclusion, this is the first study to assess angiogenesis, proliferating ECs, HIF-1*α*, VEGF and TF in each stage of the hyperplasia, *in situ* and invasive breast carcinoma sequence in a cohort of patients. These data suggest that the angiogenic switch occurs at the onset of hyperplasia in the mammary milk duct before any morphological evidence of atypia, although the greatest increase in angiogenesis occurs between *in situ* and invasive disease. Hypoxia-inducible factor-1*α*, VEGF and TF expression are significantly associated with increased angiogenesis and proliferating ECs. Moreover, a significant association between VEGF and TF in both ECs and invasive tumour cells suggests the importance of both these factors in breast cancer angiogenesis, disease progression and tumour biology. Further work is needed to examine the precise relationship between VEGF and TF in breast carcinogenesis, and to establish whether TF has a key independent role in tumour angiogenesis and disease progression that is functionally distinct from the role in VEGF production.
